# Quantitative Crotonylome Analysis Reveals the Mechanism of Shenkang Injection on Diabetic Nephropathy

**DOI:** 10.1155/2022/7767431

**Published:** 2022-07-12

**Authors:** Min Wang, Bin Zhang, Chenyang Zhang, Xuelian Zhang, Shuang Tang, Guibo Sun, Xiaobo Sun

**Affiliations:** ^1^Beijing Key Laboratory of Innovative Drug Discovery of Traditional Chinese Medicine (Natural Medicine) and Translational Medicine, Institute of Medicinal Plant Development, Chinese Academy of Medical Sciences & Peking Union Medical College, Beijing 100193, China; ^2^Key Laboratory of New Drug Discovery Based on Classic Chinese Medicine Prescription, Chinese Academy of Medical Sciences, Beijing 100193, China; ^3^NMPA Key Laboratory for Research and Evaluation of Pharmacovigilance, Beijing 100193, China

## Abstract

Shenkang injection (SKI) has been widely used in the clinical treatment of chronic kidney diseases in China because of its efficacy and safety. However, the underlying mechanism of SKI in diabetic nephropathy (DN) remains unclear. The present study aimed to investigate the renoprotective effects and possible mechanisms of SKI in diabetic db/db mice. We showed that SKI ameliorated hyperglycemia and abnormal renal biochemical parameters in db/db mice. Crotonylome and subsequent bioinformatics analyses indicated that the molecular functions of the significantly different crotonylated proteins regulated by SKI were closely related to oxidoreductase activity and oxidative phosphorylation might be one of the main pathways through which SKI functions in DN. Subsequent PRM validation of the selected crotonylated proteins confirmed these findings. In addition, we determined that SKI could regulate the expression of specific proteins in oxidative phosphorylation complexes and enhance antioxidant capacity. Taken together, our data suggest that SKI exerted the protective effect against DN potentially through reversing the abnormal crotonylation expression of oxidoreductase-related proteins.

## 1. Introduction

Diabetic nephropathy (DN) is a severe microvascular complication of diabetes and has become a major cause of chronic kidney disease (CKD) and end-stage renal disease (ESRD) [1]. Although current therapeutic strategies for DN targeting the fundamental dysregulation of the glycemia and renin-angiotensin system have been shown to delay the progression of DN, their efficacy is unsatisfactory, and the morbidity associated with DN remains high [2]. Thus, it is urgent to develop more effective therapeutic approaches for patients with DN.

Both clinical and experimental studies have suggested that hyperglycemia-induced oxidative stress is a crucial pathogenic mechanism that leads to kidney damage in DN, which is caused by the generation of excess reactive oxygen species (ROS) in response to hyperglycemia. ROS overproduction further results in oxidative damage to mitochondrial constituents (DNA, proteins, and lipids) that leads to mitochondrial dysfunction and cell death in the diabetic kidney [3, 4]. Mitochondrial respiratory chain complexes, through the oxidative phosphorylation (OXPHOS) pathway, are considered the main cellular source of ROS under normal and pathological conditions [5]. Reports show increased activities and protein levels of complexes I and III in the kidneys of diabetic mice [6] and decreased activity of renal complex III in streptozotocin (STZ-)-induced diabetic rats [7]. Further studies demonstrated that the nonconcordance of each complex in the renal mitochondria is related to an excess of ROS production and oxidative damage in diabetic db/db mice [8]. Thus, interrupting mitochondrial complexes in diabetic kidneys to limit ROS generation may be a promising therapeutic strategy to ameliorate renal damage. However, the precise underlying molecular mechanisms of mitochondrial complex-generated ROS in DN remain to be fully elucidated.

In diabetic kidneys, several protein posttranslational modifications (PTMs) have been shown to modulate mitochondrial protein integrity and function, including acetylation, advanced glycation, nitrosylation1, and ubiquitination [9, 10]. Except for these PTMs, the role of other types of PTMs remains unclear. Lysine crotonylation (Kcr) is a recently recognized modification that exists in mammals and is thought to be important for various biological processes [11]. Previous studies have demonstrated that Kcr in renal tissue increases during experimental acute kidney injury (AKI), and crotonate administration prevented a decrease in renal function [12]. Moreover, reports indicate that Kcr is maintained at high levels in chronic renal failure patients [13], while crotonylation modification was reduced in hemodialysis patients [14]. On this basis, we speculate that modulation of Kcr levels plays a crucial role in kidney injury, and it may be closely related to the pathogenesis of DN, which might provide a new approach for the treatment of DN.

Shenkang injection (SKI; drug approval number Z20040110), which was developed from the extracts of four medicinal plants: radix etrhizoma rhei, radix astragali, radix salviae miltiorrhizae, and Flos carthami, was approved for clinical treatment of CKD (Permission No. YBZ08522004) by the China Food and Drug Administration in 1999 [15]. Clinical studies showed that SKI can improve clinical efficacy in patients with early DN [16, 17]. Moreover, our previous studies demonstrated that SKI protects against DN in STZ-induced mice by enhancing antioxidant and anti-inflammatory activities [18]. Nevertheless, whether SKI is potentially renoprotective in DN and its potential mechanisms requires further analysis.

Herein, we investigated the effectiveness of SKI against DN in db/db mice and elucidated the underlying mechanism of SKI by integrating TMT-based quantitative crotonylome approach, bioinformatics analysis, parallel reaction monitoring (PRM) for targeted quantitation, and experimental validation. Our work provides a landscape of crotonylation in kidney of diabetic mouse upon SKI treatment and promotes the understanding of protective mechanism of SKI against DN from a new perspective.

## 2. Materials and Methods

### 2.1. Animal Experiments

All animal experiments were approved by the Laboratory Animal Ethics Committee of the Institute of Medicinal Plant Development, Peking Union Medical College, and conformed to the Guide for the Care and Use of Laboratory Animals published by the US National Institutes of Health (NIH Publication, 8th Edition, 2011).

Six-week-old male C57BLKS/J diabetic mice (db/db) and their nondiabetic littermates (db/m) were purchased from the Model Animal Research Center of Nanjing University (Nanjing, China). Mice were socially housed (2-3 mice per cage) at a constant temperature of 22°C ± 2°C, 40-60% humidity, and a 12-h light and 12-h dark cycle. All animals were given standard rodent chow and water ad libitum. After 8 weeks of adaptation, db/db mice with fasting blood glucose (FBG) >13.9 mmol/L were considered diabetic and used for further experimentation [19]. The mice were then randomly assigned to five groups (*n* = 15): db/m mice treated with vehicle (db/m), db/db mice treated with vehicle (db/db), db/db mice treated with 7.5 ml/kg SKI (db/db+SKI-L), db/db mice treated with 15 ml/kg SKI (db/db+SKI-M), and db/db mice treated with 30 ml/kg SKI (db/db+SKI-H). SKI (TYZ20181001) was supplied by Shijishenkang Co., Ltd. (Xi'an, China). The SKI dose was based on clinical equivalent dose and our previous animal studies [18]. SKI was injected intraperitoneally every day for 6 weeks. The mice in the db/m and db/db model groups were given the same volume of vehicle (saline solution).

### 2.2. Blood and Urine Examination

After the 6-week experimental period, FBG levels were measured with a glucose meter (One Touch Ultra, Lifescan, USA). 24-hour urine was obtained using a metabolic cage without food supply a day before sacrifice. Urinary albumin levels were determined by kits from the Nanjing Jiancheng Bioengineering Institute (Nanjing, China). Blood samples were gathered from each animal via retro-orbital puncture before sacrifice. Serum creatinine (Scr) and blood urea nitrogen (BUN) levels were measured using commercially available kits (Jiancheng Bioengineering Institute, Nanjing, China) according to the manufacturer's instructions.

### 2.3. TMT-Based Crotonylation Quantitative Proteomics

The harvested kidneys of db/m, db/db, and 30 ml/kg SKI-treated db/db mice (db/db + SKI) groups were immediately stored in liquid nitrogen. Sample preparation, TMT labeling, Kcr peptide enrichment, and high-resolution liquid chromatography tandem mass spectrometry (LC-MS/MS) analysis were conducted by Jingjie PTM BioLab Co. Ltd. (Hangzhou, China). Detailed procedures are described in the Supplemental Information.

### 2.4. Database Search

The resulting MS/MS data were processed using the MaxQuant search engine (v.1.5.2.8) [20]. False discovery rate (FDR) values were set at less than 1%, and the minimum score for modified peptides was set at >40. The site localization probability was set to >0.75. Detailed procedures are described in the Supplemental Information.

### 2.5. Bioinformatics Analysis

The UniProt-GOA database (http://www.ebi.ac.uk/GOA/) provided Gene Ontology (GO) annotation data. InterProScan (http://www.ebi.ac.uk/interpro) with the help of the protein sequence alignment method was used to annotate the protein structures domain functions. The KEGG database (http://www.genome.jp/kegg/) was used to identify protein pathways. A corrected *P* value of <0.05 was considered significant. Subcellular localization was predicted in WoLFPSORT database (https://www.genscript.com/wolf-psort.html). A *P* value <0.05 between comparative groups was defined as differentially expressed proteins. Cluster analysis was visualized by R studio (v. 1.3.1073) and heat map gplots package. The iceLogo (https://iomics.ugent.be/icelogoserver/) was used to analyze the sequence motif of Kcr sites using a *P* value of 0.05. The protein-protein interaction (PPI) network was determined based on the STRING database (http://string-db.org/) and visualized using Cytoscape (v.3.7.2).

### 2.6. Parallel Reaction Monitoring Analysis

Protein isolation and trypsinization were conducted as described above, and peptide samples were dissolved in 0.1% formic acid and injected into an easy-nLC 1200 (Thermo Fisher Scientific, Waltham, MA, USA) UPLC system [21]. Detailed procedures are described in the Supplemental Information.

### 2.7. Isolation of Mitochondria

Mitochondria from mouse kidneys were isolated using a MinuteTM mitochondria isolation kit (MP-007, Invent Biotechnologies, Plymouth, MN, USA) according to the manufacturer's instructions. Detailed procedures are described in the Supplemental Information. The isolated mitochondria samples were then stored at -80°C for western blot analyses with the indicated antibodies.

### 2.8. Western Blotting Analysis

Tissue lysate preparation and western blot (WB) analysis were performed as previously described [22]. The primary antibodies used were as follows: total OXPHOS antibody cocktail (ab110413, Abcam, Cambridge, UK), VDAC (4661S, Cell Signaling Technology, USA), glutathione peroxidase 3 (sc-58361, Santa Cruz Biotechnology, USA), and GAPDH (YM3029, Immunoway, China). Specific bands were visualized by enhanced chemiluminescence.

### 2.9. Immunoprecipitation (IP)

Whole kidney tissue lysates were incubated with rabbit anti-COX5A (ab180129, Abcam, Cambridge, UK), rabbit anti-Ndufs4 (ab137064, Abcam, Cambridge, UK), mouse anti-Gpx3 (sc-58361, Santa Cruz Biotechnology, USA), or rabbit control IgG (FW20532, BEIJING TDR BIOTECH CO., LTD, China) with gentle rocking overnight at 4°C, followed by immunoprecipitation using protein A/G agarose beads (SA032050, Smart-Lifesciences, China). The bound proteins were eluted with loading buffer, and the elution was analyzed by WB analysis with anticrotonyllysine antibody (PTM-502, Jingjie PTM BioLab Co. Ltd, China), as described previously [22].

### 2.10. Measurement of GPx Activity

Activity of glutathione peroxidase (GPx) was spectrophotometrically measured using an assay kit from Nanjing Jiancheng bioengineering Institute (Nanjing, China) according to the manufacturer's instructions.

### 2.11. Statistical Analysis

Results are expressed as mean ± standard error of the mean (SEM) of three independent experiments. Comparisons between two groups were performed by Student's *t*-test, while one-way analysis of variance with Tukey's post hoc test was used for multigroup comparisons. Statistical significance was set at *P* < 0.05.

## 3. Results

### 3.1. SKI Ameliorates Renal Injury in Type 2 Diabetic Mice

**Figure 1:**
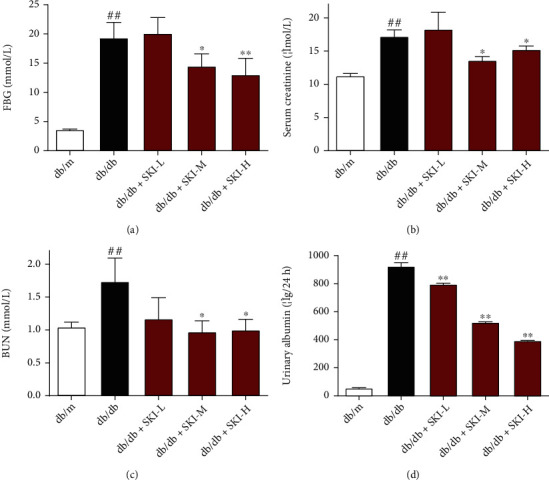
Shenkang injection ameliorated biochemical characteristics of renal function in db/db mice. (a) Fasting blood glucose levels were measured at the end of the experiment. (b) Detection of serum creatinine levels in mice. (c) Blood urea nitrogen levels in investigated mice. (d) Urinary albumin excretion of mice in 24 hr. Data are expressed as the mean ± standard error of mean. #*P* < 0.05 and ##*P* < 0.01 versus db/m mice; ∗*P* < 0.05 and ∗∗*P* < 0.01 versus db/db mice. (*n* = 10 mice in each group).

The db/db mouse, a classic animal model of type 2 diabetes [23], was used to investigate the renal protective effects of SKI. The db/db mice show significantly elevated levels of FBG at the end of the experiment, as shown in Figure 1. In contrast, FBG levels were improved by SKI compared to those in db/db mice. Scr and BUN levels were significantly higher in db/db mice than in db/m mice, suggesting a severe impairment of renal function. SKI treatment triggered a significant reduction in Scr and BUN levels in db/db mice. Moreover, urinary albumin is considered a key marker for evaluating the progression of DN [23]. Urinary albumin was drastically elevated in db/db mice and was markedly decreased in SKI-treated db/db mice.

### 3.2. Profiles of Kcr Proteins and Sites in Kidney Tissues of db/m, db/db, and SKI-Treated db/db Mice

**Figure 2:**
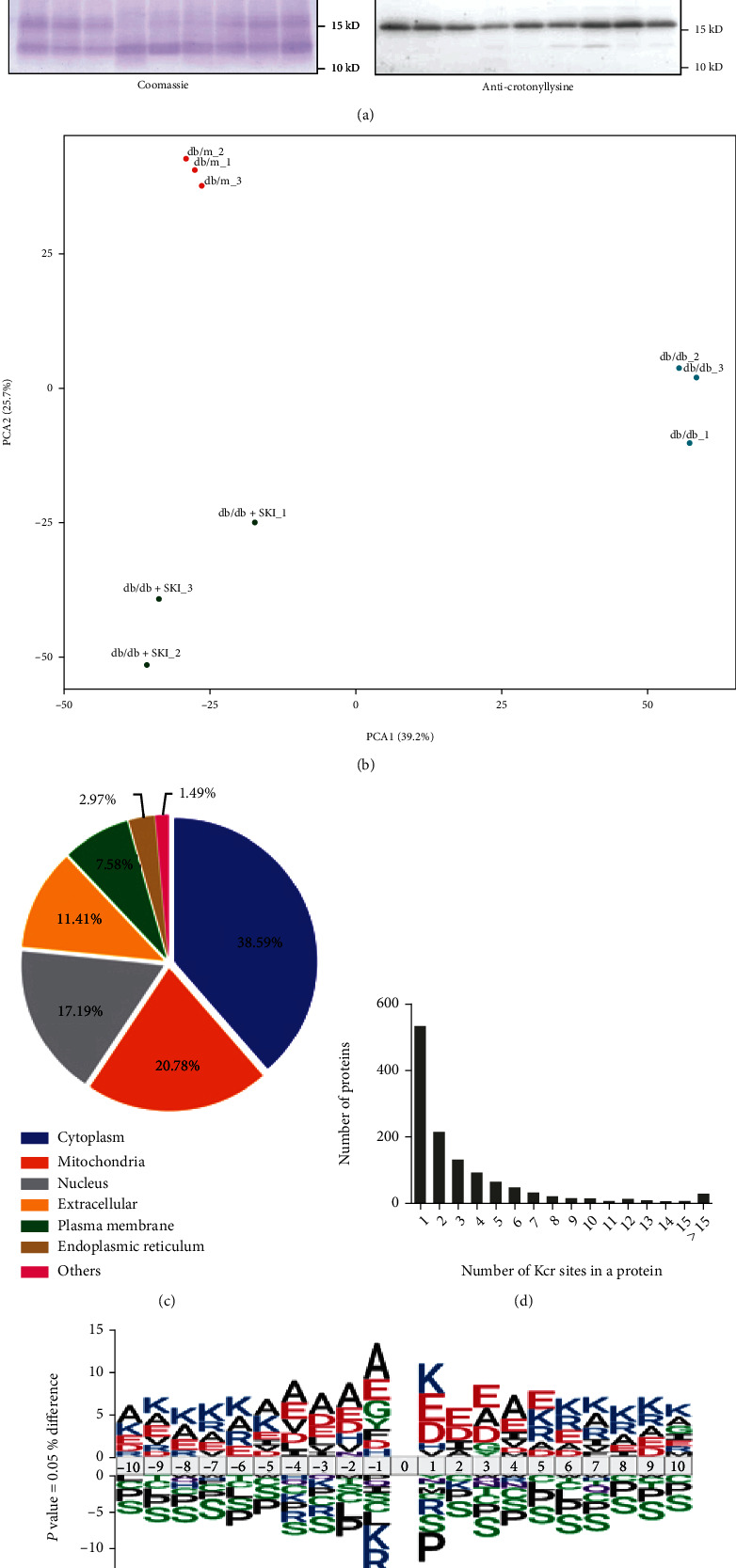
Global analysis of crotonylation proteome in the kidney of db/m, db/db and Shenkang injection (SKI-)-treated db/db mice. (a) Western blot analysis of kidney tissue lysates from db/m, db/db, and SKI-treated db/db mice probed with anticrotonyllysine antibody (*n* = 3). Equal loading was verified using Coomassie blue staining. (b) Principal component analysis (PCA) of the crotonylome in the db/m, db/db, and SKI-treated db/db mice groups (*n* = 3). (c) Subcellular localization prediction of Kcr proteins in kidney tissues. (d) The number of the identified crotonylated sites per protein. (e) Sequence logo of Kcr sites determined by iceLogo.

The existence and differences in Kcr protein modification were investigated by western blotting with anticrotonyllysine antibody in three kidney tissue lysates of db/m, db/db, and 30 ml/kg SKI-treated db/db mice (db/db+SKI) (Figure 2(a)). Equal loading was verified using Coomassie blue staining. To identify and quantify differentially expressed crotonylated lysine sites and proteins, the lysine-crotonyl proteomics (crotonylome) of kidney samples from three groups (db/m, db/db, and db/db+SKI mice) were analyzed using TMT labeling crotonylation quantitative proteomic techniques (Figure S1 in Supporting Information). The mass error distribution was near zero (Figure S2(a) in Supporting Information), and the length of most peptides ranged from 7 to 23 amino acids (Figure S2(b) in Supporting Information). Altogether, we identified and quantified a total of 4200 Kcr sites from 1229 proteins among the three groups (Figure S2(c) in Supporting Information). Principal component analysis (PCA) demonstrated that the protein pattern from the three different groups distinguished well (Figure 2(b)).

The subcellular location analysis of differentially crotonylated proteins revealed that these Kcr proteins exert widespread cellular impact in multiple compartments (Figure 2(c)). Among these Kcr proteins, 532 (43.36%) contained a single site, whereas 28 Kcr proteins had >15 Kcr sites (Figure 2(d)). To further determine the sequence characteristics around the Kcr sites, iceLogo [24] was applied to analyze the amino acids sequences surrounding the identified Kcr sites. Evident enrichment of positively charged lysine, negatively charged amino acids, glutamic acid, and aspartic acid were found at the +1 position of Kcr sites (Figure 2(e)). Consistently, motif analysis using Motif-X algorithms identified AKcrK and AKcrE motifs as the most conserved sequences for Kcr sites (Table S1 in Supporting Information).

### 3.3. Analysis of the Differential Kcr Sites and Proteins

To understand the function of Kcr in the pathogenesis of DN, we compared the differential Kcr levels between the three groups (db/db vs. db/m, db/db+SKI vs. db/db, and db/db+SKI vs. db/m). The differential changes in Kcr were normalized to the total protein expression levels. A change threshold of at least 1.3 times and a *t*-test with *P* < 0.05 were recognized as a standard to evaluate the differences among the three groups. As shown in Table 1, 554 Kcr sites in 383 proteins are increased, and 366 Kcr sites in 216 proteins are decreased in the db/db vs. db/m group. A total of 247 Kcr sites in 148 proteins were increased, and 567 Kcr sites in 406 proteins were decreased in the db/db+SKI vs. db/db group.

**Figure 3:**
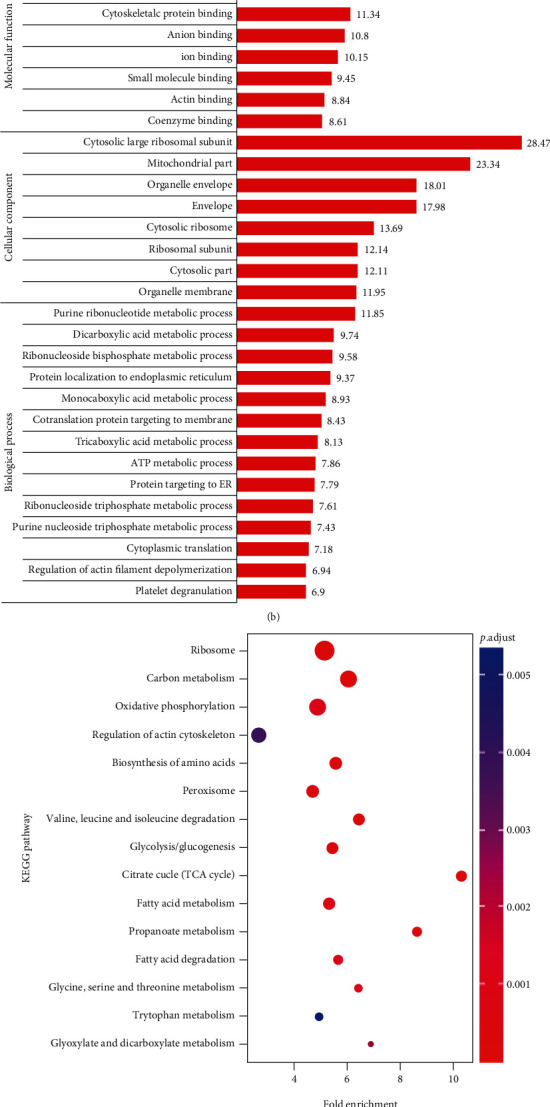
Differential crotonylation proteins and biological pathways identified to be regulated by Shenkang injection (SKI). (a) Cluster analysis of significantly changed Kcr proteins in db/m, db/db, and SKI-treated db/db mice groups according to their change tendencies (each fold line represents a protein). Proteins in clusters 1 and 3 were regarded as regulated by SKI. (b) Gene Ontology (GO) enrichment analysis of Kcr proteins regulated by SKI. (c) KEGG pathway enrichment analysis of Kcr proteins regulated by SKI. (d) Protein-protein interaction (PPI) network analysis for the Kcr proteins regulated by SKI based on the STRING database.

To explore the significant changes in crotonylation regulated by SKI treatment, we performed cluster analysis in the three groups. The differential levels of crotonyl peptides were divided into four clusters according to their changing tendencies (Figure 3(a)). Kcr proteins in cluster 1 were significantly elevated in the db/db group but reduced in the SKI-treated group. Kcr proteins in cluster 3 displayed significant decrease in the db/db group and increase in the SKI-treated group. Proteins in clusters 2 and 4 were not obviously affected by SKI. According to these results, Kcr proteins in clusters 1 and 3 were regarded as modulated by SKI. The molecular functions of these Kcr proteins in clusters 1 and 3 were mainly associated with oxidoreductase activity via GO analysis (Figure 3(b)). KEGG pathway analysis showed enrichment in terms related to ribosomes, carbon metabolism, and OXPHOS (Figure 3(c)). Using PPI network analysis via molecular complex detection (MCODE) on the differentially expressed crotonylated proteins in clusters 1 and 3, we identified two highly interactive modules which involved in the ribosome and OXPHOS (Figure 3(d)).

### 3.4. Validation of Crotonylation by PRM

**Figure 4:**
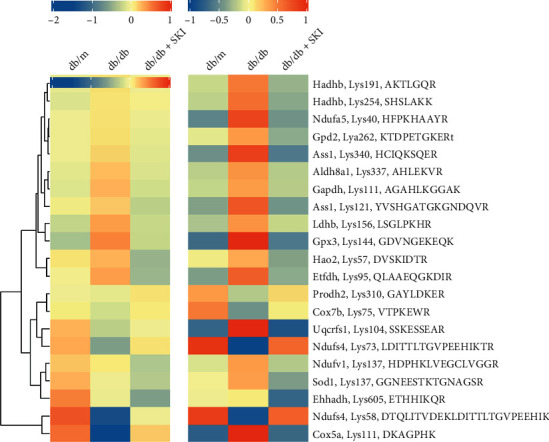
Heatmap summarizing temporal profiles of 21 selected crotonyl peptides. Parallel reaction monitoring (left) and tandem mass tag-based (right) temporal crotonylation profiles are depicted (*n* = 3).

To confirm the Kcr protein levels acquired by TMT analysis, 21 differentially abundant Kcr sites in the kidneys of db/m, db/db, and SKI-treated db/db mice groups were selected based on the crotonyl proteomics data and further quantified by the PRM assay. We compared the PRM analysis results with those from the TMT data and found a good correlation (Figure 4). However, some Kcr sites showed a poor correlation between TMT and PRM data, such as Cox5aLys111.

**Figure 5:**
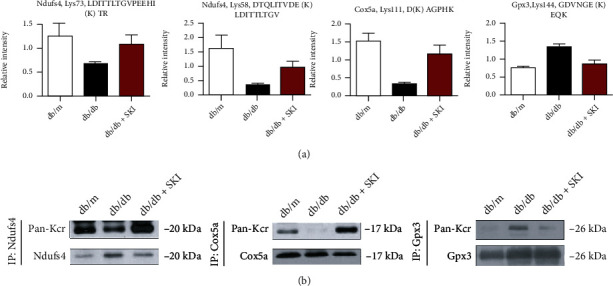
Validation of significantly differential crotonylation proteins regulated by Shenkang injection (SKI) in db/db mice. (a) Three potential crotonylation proteins were validated in kidney tissues from db/m, db/db, and SKI-treated db/db mice using parallel reaction monitoring (PRM). (b) Three indicated Kcr proteins that showed a significant difference in SKI-treated db/db mice compared with db/db mice by PRM method and with commercially available antibodies available for immunoprecipitation were selected. Kidney tissue lysates were immunoprecipitated by the selected antibodies and protein A agarose beads followed by immunoblotting with anti-Pan Kcr. Data are expressed as the mean ± standard error of mean from three independent experiments. #*P* < 0.05 versus db/m mice; ∗*P* < 0.05 versus db/db mice.

Using the PRM strategy, we observed three novel crotonylation sites of the proteins (NADH:ubiquinone oxidoreductase subunit S4, Ndufs4; cytochrome c oxidase subunit 5a, Cox5a; and glutathione peroxidase 3, Gpx3) that are associated with the regulation of oxidative phosphorylation. The relative abundance data of crotonyl peptides from the three individual proteins are presented in Figure 5(a). The Kcr levels of lysine 73 on Ndufs4 and lysine 111 on Cox5a were decreased in db/db mice, and they were increased in SKI-treated db/db mice. The Kcr levels of lysine 144 on Gpx3 were increased in db/db mice and were reduced to normal levels in SKI-treated db/db mice. To investigate the position of the novel identified Kcr sites in protein secondary structures, we labeled the Kcr sites in the three-dimensional crystal structures of Ndufs4, Cox5a, and Gpx3 (Figure S3 in Supporting Information).

### 3.5. Verification of Lysine Crotonylation by Immunoprecipitation Coupled with Western Blot Analysis

We immunoprecipitated equal amount of proteins (Ndufs4, Cox5a, and Gpx3) from db/m, db/db, and db/db+SKI samples, respectively, and the Kcr levels were detected by WB with anti-PAN Kcr antibodies. Consistent with the same Kcr trend suggested by the PRM analysis, the Kcr levels of Ndufs4 and Cox5a proteins were lower in db/db kidneys than in db/m kidneys, but SKI+db/db mice showed strongly augmented crotonylation levels (Figure 5(b)). In contrast, the db/db mice group showed higher Kcr levels of Gpx3 compared with the db/m mice group, which could be diminished by SKI treatment.

### 3.6. SKI Regulates the Kcr Level Associated with Enhanced Antioxidant Capacity

**Figure 6:**
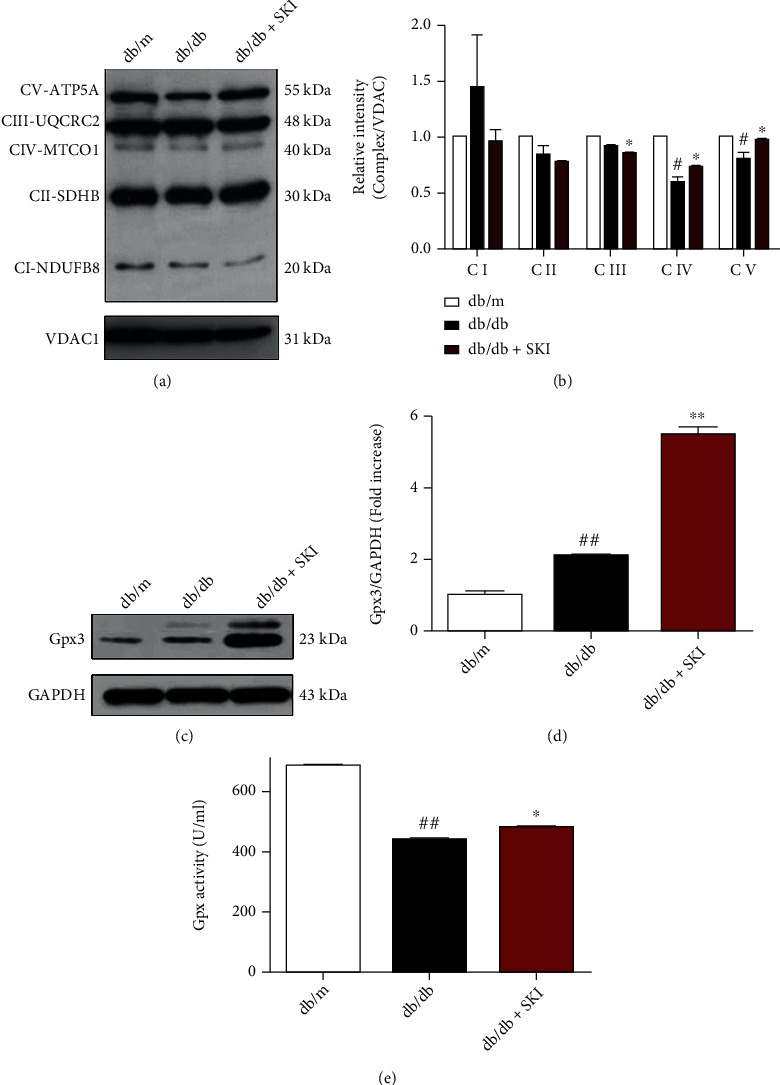
Protective effects of Shenkang injection (SKI) against diabetic nephropathy associated with regulation of oxidative phosphorylation (OXPHOS). (a) Western blot analysis showing the effect of SKI on renal mitochondria OXPHOS complex expressions in db/db mice. (b) Quantified band densities for complex I-V in db/m, db/db, and SKI-treated db/db mice renal mitochondria. (c) Western blot detects the protein expression levels of Gpx3 in the kidney tissues. GAPDH was used as the loading control. (d) Quantification of Gpx3 levels relative to GAPDH. (e) Plasma glutathione peroxidase (GPx) activity. Data are expressed as the mean ± standard error of mean from three independent experiments. #*P* < 0.05 and ##*P* < 0.01 versus db/m mice; ∗*P* < 0.05 and ∗∗*P* < 0.01 versus db/db mice.

We hypothesized that the Kcr levels of the Ndufs4, Cox5a, and Gpx3 proteins regulated by SKI are correlated with the induction of antioxidant defenses. Results obtained from WB analysis showed that SKI significantly regulated the expression of mitochondrial complexes IV and V in the kidneys of db/db mice (Figures 6(a) and 6(b)), suggesting that SKI improves oxidative OXPHOS in the diabetic kidney by regulating the kidney mitochondrial respiratory chain subunit complexes. Moreover, the expression level of Gpx3 protein was markedly upregulated in the db/db+SKI group compared with the db/db group (Figures 6(c) and 6(d)). In addition, we measured the activity of the plasma antioxidant enzyme GPx. It was found that the GPx activity in the db/db group was significantly decreased, while SKI significantly promoted the increase in GPx activity (Figure 6(e)). These findings indicate that SKI might modulate OXPHOS complex expression as well as the antioxidant activity of enzymes to maintain oxidative homeostasis.

## 4. Discussion

Crotonylation was first identified in histones and has been recognized in kidney tissues as a crucial contributor to the epigenetic regulation [25]. Recent advances in lysine crotonylation have exhibited that numerous nonhistone proteins are frequently modified by crotonylation, and they are involved in many biological processes such as cellular metabolism, cell cycle, and cellular organization processes [26]. In the current study, we carried out crotonyl proteomics to further investigate the potential mechanism in kidney tissues of SKI-treated type 2 diabetic mice. We quantified a total of 4200 Kcr sites from 1229 proteins in kidney tissues of all three groups and found that Kcr proteins were largely located in the cytoplasm, mitochondria, nucleus, and extracellular space, which demonstrated that crotonylation may take place in a diversity of cellular proteins and may affect many biological processes in the kidney tissues of db/db mice. Among them, 148 up and 406 downregulated Kcr proteins of more than ±1.3 fold change were identified in the db/db + SKI versus db/db group. These results indicate a putative association between the renal protective effects of SKI and Kcr in DN.

Augmented oxidative stress caused by the overproduction of ROS relative to antioxidant defenses has been implicated in the development of DN [3]. In our previous studies, we found that SKI protects against DN in STZ-induced mice by enhancing antioxidant activities [18]. Bioinformatics analysis aligns well with previous studies, which revealed that the molecular functions of the altered Kcr proteins regulated by SKI were enriched in oxidoreductase activity. We additionally identified that these Kcr proteins interacted with each other and were involved in ribosome functions and OXPHOS via PPI analysis. Besides, there were two pathways closely associated with the “OXPHOS” pathway, including “citrate cycle” and “glycolysis/gluconeogenesis.” Intracellular ROS are produced through the OXPHOS pathway in response to hyperglycemia [27]. OXPHOS occurs in the mitochondrial electron transport chain (ETC) located within the mitochondrial matrix [28]. Mitochondrial ETC is composed of five complexes (I-V). Complexes I-IV carry out a series of redox reactions, and complex V is in charge of ATP production. During this process, ROS are generated for signaling and cause cellular injury under physiological and pathological states [28]. Accumulating evidence has demonstrated that ETC disruption contributes to increased ROS and oxidative damage, which might be important in the pathogenesis of DN [5]. Here, we found that multiple ETC complex proteins were crotonylated using proteomic analysis of Kcr in diabetic kidneys, and western blot results demonstrated that SKI significantly regulated the protein levels of renal ETC subunit complexes in db/db mice. These observations implied that crotonylated ETC complex proteins regulated by SKI may play a critical role in the electron transfer cascade and redox homeostasis in diabetic kidneys.

**Figure 7:**
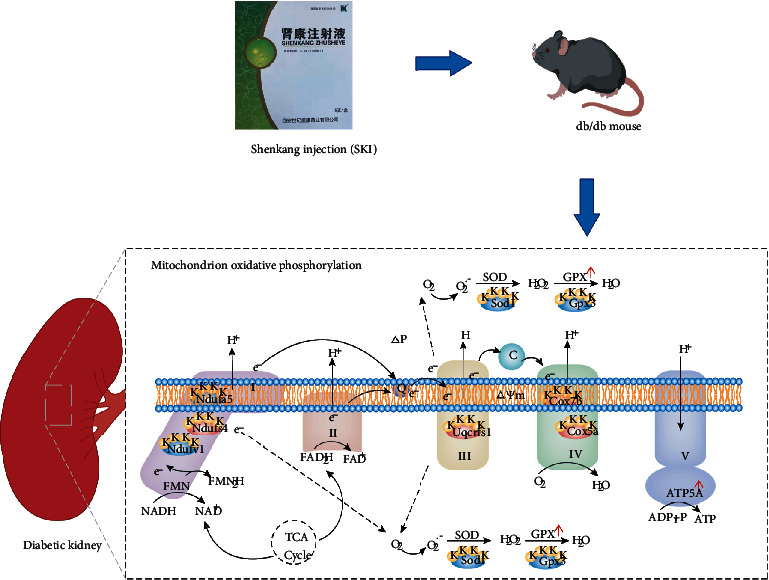
Schematic diagram of differentially expressed crotonylated proteins regulated by Shenkang injection (SKI) against diabetic renal damage in oxidative phosphorylation pathway. The yellow circular shape with K depicts crotonylated lysine residues. The oval with light red and blue color indicates crotonylation proteins upregulated and downregulated by SKI in oxidative phosphorylation, respectively.

The verification of often unexplored crotonylation sites is tough due to the lack of site-specific crotonylation antibodies. The quantitative MS approach, namely, PRM, is a widely used independent technique for initial validation [21, 29]. PRM, which is regarded as an antibody-free but MS-based western blot, is capable of the specific quantification of only a restricted amount of selected targets with greatly higher precision and accuracy [30]. Based on our TMT results, we selected a subset of crotonylation sites that are associated with the regulation of redox homeostasis and energy metabolism for further targeted analysis by PRM to better assess whether they involve in the regulation of the diabetic kidney by SKI. Our results showed that the TMT and PRM results were highly consistent. According to our results (Figure 7), some of the Kcr sites involved in the ETC complex were upregulated by SKI treatment, such as K73 on Ndufs4 accessory subunit of complex I [31] and K111 on Cox5a subunit of complex IV [32]. However, Kcr sites that are related to antioxidant enzymes undergo downregulation upon SKI treatment, such as K144 on Gpx3 (Figure 7). The Kcr levels of Ndufs4, Cox5a, and Gpx3 were also verified by immunoprecipitation and western blot experiments, which were consistent with the PRM results. These results validate that SKI reversed the abnormal Kcr expression of redox homeostasis-related proteins in the kidneys of diabetic mice.

GPx3, also called plasma GPx, is a selenoprotein antioxidant enzyme that is mainly produced by the kidney proximal tubule epithelial cells, which is secreted and dispersed to the plasma, circulating platelets, and endothelial microenvironment [33]. Loss of GPx3 has been reported in patients with CKD undergoing hemodialysis [34, 35]. Besides, GPx-3 activity seems to have an opposite correlation with the decline of glomerular filtration rate in diabetes and advanced CKD patients [36]. The upregulation of GPx-3 expression is permissively cytoprotective in experimental studies of tissue injury [36, 37]. Neutralization of ROS by this enzyme is related to decreased glucose-dependent kidney and vascular damage, which suggests that GPx3 exerts an crucial role against oxidative stress and it may act as a potential therapeutic target for diabetic kidney disease [35, 36]. Despite the importance of Gpx3 in DN, little is known regarding its regulation. Our results demonstrated that SKI treatment decreased the Kcr level of Gpx3 and promoted both Gpx3 expression and GPx activity in db/db mice, indicating that downregulation of Gpx3 crotonylation by SKI in diabetic kidneys is correlated with increased expression and activity. These results also suggest that the modified Kcr sites may directly affect activity or other domains which could affect protein-protein interactions or subcellular localization of Gpx3. However, further studies are required to interpret the functional consequences of Kcr on Gpx3.

To further identify the bioactive compounds responsible for the observed effects of SKI, seven bioactive constituents, including gallic acid, danshensu, hydroxysafflor yellow A, calycosin, aloe-emodin, rhein, and emodin, were selected based on tissue distribution and pharmacokinetic studies of SKI [38–40] and then analyzed for molecular docking with Gpx3 around its K144 site. The docking results showed that these bioactive constituents, except hydroxysafflor yellow A, could bind with Gpx3 (Table S2 in Supporting Information). The binding modes of emodin, rhein, aloe-emodin, calycosin, danshensu, and gallic acid with Gpx3 are shown in Figure S4 in Supporting Information. Our study suggests that SKI enhances antioxidant activity, which is probably achieved by targeting Gpx3. However, the bioactive compounds in SKI and their actions need to be explored in future studies.

## 5. Conclusions

In summary, our comprehensive analysis of lysine crotonylation in kidney tissues of db/m, db/db, and SKI-treated db/db mice provides a novel understanding of the protective role of SKI against DN. PRM and biological verification indicated that SKI reversed the abnormal Kcr expression of oxidoreductase-related proteins in the kidneys of diabetic mice, thereby enhancing antioxidant capacity. This study not only broadens our understanding of the mechanism by which SKI alleviates renal injury in diabetic mice but also provides new evidence for the important roles of crotonylation in diabetic kidney disease.

## Figures and Tables

**Table 1:** Statistics of the differential expressed Kcr sites and proteins.

	Type	db/db vs. db/m	db/db+SKI vs. db/db	db/db+SKI vs. db/m
Up	*Kcr site*	*554*	*247*	*164*
	*Kcr protein*	*383*	*148*	*98*

Down	*Kcr site*	*366*	*567*	*205*
	*Kcr protein*	*216*	*406*	*142*

## Data Availability

Data are available upon reader's request.

## Abbreviations

BUN: Blood urea nitrogen
CKD: Chronic kidney disease
Cox5a: Cytochrome c oxidase subunit 5a
DN: Diabetic nephropathy
FBG: Fasting blood glucose
Gpx-3: Glutathione peroxidase 3
IP: Immunoprecipitation
Kcr: Crotonylation
LC-MS/MS: Liquid chromatography tandem mass spectrometry
Ndufs4: NADH:ubiquinone oxidoreductase subunit S4
OXPHOS: Oxidative phosphorylation
PCA: Principal component analysis
PPI: Protein-protein interaction
PRM: Parallel reaction monitoring
Scr: Serum creatinine
SKI: Shenkang injection
ROS: Reactive oxygen species
TMT: Tandem mass tag
WB: Western blot.

## Supplementary Materials

Supplementary 1 Supplemental Methods: Detailed procedures of TMT-based crotonylation quantitative proteomics, database search, parallel reaction monitoring analysis, and isolation of mitochondria.

Supplementary 2 Supplementary Table S1: Motif analysis of Kcr peptides.

Supplementary 3 Supplementary Table S2: Molecular docking results of Gpx3 and bioactive constituents of Shenkang injection.

Supplementary 4 Supplementary Figure S1: Schematic representation of the experimental workflow for tandem mass tag quantification of Kcr in kidney tissues from db/m, db/db, and Shenkang injection-treated db/db mice.

Supplementary 5 Supplementary Figure S2: Verification of the mass spectrometry (MS) data.

Supplementary 6 Supplementary Figure S3: The crystal structures of three crotonylated proteins: Ndufs4, cox5a, and Gpx3.

Supplementary 7 Supplementary Figure S4: The 3D interaction diagrams of Gpx3 and bioactive constituents of Shenkang injection.
