# Supplementary material for: Quantitative Crotonylome Analysis Reveals the Mechanism of Shenkang Injection on Diabetic Nephropathy
Source: Oxid Med Cell Longev. 2022 Jul 12;2022:7767431. doi: 10.1155/2022/7767431 (PMC11401665; doi:10.1155/2022/7767431)
Supplement: Supplementary 1 — Supplemental Methods: Detailed procedures of TMT-based crotonylation quantitative proteomics, database search, parallel reaction monitoring analysis, and isolation of mitochondria. [file 7767431.f1.pdf]

Supplemental Information

Supplemental Methods

TMT-based crotonylation quantitative proteomics

The harvested kidneys were immediately stored in liquid nitrogen. TMT labeling crotonylation quantitative proteomics analysis was performed by Jingjie PTM BioLab Co. Ltd (Hangzhou, China). In brief, tissues were homogenized and lysed on ice using lysis buffer supplemented with 8 M urea, 1% protease inhibitor cocktail, 3 μM trichostatin A, and 50 mM nicotinamide, followed by sonication three times using a high-intensity ultrasonic processor (Scientz). The supernatants were collected after centrifugation at 12,000 × g at 4 ℃ for 10 min. After digestion with trypsin, peptides were desalted using a Strata X C18 SPE column (Phenomenex), vacuum-dried, labeled with a TMT kit (ThermoFisher Scientific, Waltham, MA, USA), and fractionated by high-pH reverse-phase HPLC using an Agilent 300Extend C18 column (5 μm particles, 4.6 mm ID, 250 mm length).

For Kcr enrichment, Kcr-containing peptides were dissolved in pH 8.0 NETN buffer (100 mM NaCl, 1 mM EDTA, 50 mM Tris-HCl, and 0.5% NP-40) and were incubated with pre-washed anti-Kcr antibody-conjugated agarose beads (PTM Biolabs, Hangzhou, China) overnight at 4℃ with gentle shaking. Beads were then washed with NETN buffer, and the bound peptides were eluted with 0.1% trifluoroacetic acid. The resulting peptides were desalted with C18 ZipTips (Millipore) before LC-MS/MS analysis.

For LC-MS/MS analysis, peptides were dissolved in 0.1% formic acid (solvent A)

and peptide separation was performed using a reverse-phase analytical column (15-cm length, 75 μm inner diameter) inserted into an EASY-nLC 1000 UPLC system (Thermo Fisher Scientific, Waltham, MA, USA). Subsequently, the separated peptides were ionized using an NSI source (voltage of 2.0 kV) and then analyzed using a Q ExactiveTM Plus Hybrid Quadrupole-Orbitrap Mass Spectrometer (Thermo Fisher Scientific, Waltham, MA, USA) in triplicate. Peptides were then selected for MS/MS using an NCE setting of 30, and the fragments were detected at a resolution of 17,500. The data-dependent acquisition was used to acquire data.

Database search

The resulting MS/MS data were processed using the MaxQuant search engine (v.1.5.2.8). Tandem mass spectra data were searched against non-redundant mouse protein amino acid sequences from the UniProt database (https://www.uniprot.org/) concatenated with a reverse decoy database. Trypsin/P was specified as a cleavage enzyme, allowing up to four missing cleavages per peptide for crotonylome analysis. The mass tolerance for precursor ions was set as 20 ppm in the first search and 5 ppm in the main search, and the mass tolerance for fragment ions was set as 0.02 Da. Furthermore, carbamidomethyl on cysteine was specified as a fixed modification; oxidation of methionine, deacylation of asparagine and glutamine, acetylation on protein N-terminal, and crotonylation on lysine were specified as variable modifications. TMT-10 plex was the method for peptide quantification. False discovery rate (FDR) values were set at less than 1%, and the minimum score for modified peptides was set at >40. The site localization probability was set to >0.75.

All other parameters in MaxQuant were set to the default values.

Parallel Reaction Monitoring analysis

Protein isolation and trypsinization were conducted as described above, and peptide samples were dissolved in 0.1% formic acid and injected into an easy-nLC 1200 (Thermo Fisher Scientific, Waltham, MA, USA) UPLC system. The mobile phase buffer consisted of 0.1% formic acid in water and eluted with 0.1% formic acid (buffer A) in 90% acetonitrile (buffer B). The LC flow rate was 500 nL/min. The gradient was set as 9–25% buffer B for 36 min, 25–35% buffer B for 18 min, 35–80% buffer B for 3 min, and then held at 80% for the last 4 min. After separation, a parallel reaction monitoring (PRM) mass spectrometric analysis was performed via MS/MS with a Q ExactiveTM HF-X mass spectrometer (Thermo Fisher Scientific, Waltham, MA, USA). In all experiments, a full mass spectrum at 120,000 resolution (AGC target 3E6, 50 ms maximum injection time, m/z 260–965) was followed by 20 MS/MS scans at 15,000 resolution (AGC target 1E5, 200 ms maximum injection time), as triggered by an unscheduled inclusion list. An MS/MS isolation window of 1.4 m/z was used. Higher-energy collisional dissociation was used with a 28 eV normalized collision energy. The PRM data were processed using Skyline (version 20.2).

Isolation of mitochondria

Mitochondria from mouse kidneys were isolated using a MinuteTM Mitochondria Isolation Kit (MP-007, Invent Biotechnologies, Plymouth, MN, USA) according to the manufacturer’s instructions. Briefly, buffer A was added to the filter,

and the kidney tissue was ground within the filter using a plastic rod for 1 min and then placed on ice for 5 min. The filter cartridge was capped and centrifuged at 16,000 × g for 30 s. Discard the filter and resuspend the pellet by vortexing and centrifuge at 700 X g for 1 min, carefully transfer the supernatant to a fresh 1.5 ml tube and add 300 μl buffer B to the tube. After centrifugation at 16,000 × g for 10 min, the supernatant was collected as the cytoplasmic fraction, and the pellet was resuspended in 200 μl buffer B. After centrifugation at 8,000 × g for 5 min, the supernatant was transferred to a 2.0 ml tube to which a 1.6 ml volume of cold phosphate-buffered saline was added. The tube was centrifuged at 16,000 × g for 25 min. The supernatant was discarded and the pellet was saved (isolated mitochondria). Mitochondria samples were then stored at -80℃ for western blot analyses with the indicated antibodies.
