# Supplementary material for: Quantitative Crotonylome Analysis Reveals the Mechanism of Shenkang Injection on Diabetic Nephropathy
Source: Oxid Med Cell Longev. 2022 Jul 12;2022:7767431. doi: 10.1155/2022/7767431 (PMC11401665; doi:10.1155/2022/7767431)
Supplement: Supplementary 2 — Supplementary Table S1: Motif analysis of Kcr peptides. [file 7767431.f2.pdf]

Supplementary Table S1. Motif analysis of Kcr peptides

Motif	Motif Score	Foreground		Background		Fold Increase
		Matches	Size	Matches	Size	
xxxxxxxxxA_K_Exxxxxxxxx	22.82	83	6010	2855	516020	2.5
xxxxxxxxxA_K_Kxxxxxxxxx	26.80	90	5927	2467	513165	3.2
xxxxxxxxAx_K_xxxxxxxxxx	16.00	535	5837	32531	510698	1.4
xxxxxxxxxA_K_xxxxxxxxxx	15.16	396	5302	23517	478167	1.5
xxxxxxxxxx_K_Dxxxxxxxxx	14.96	415	4906	25716	454650	1.5
xxxxxxxxxx_K_Kxxxxxxxxx	15.99	541	4491	36167	428934	1.4
xxxxxxxxxx_K_Exxxxxxxxx	16.00	578	3950	39787	392767	1.4
xxxxxKxxxx_K_xxxxxxxxxx	14.61	374	3372	25850	352980	1.5
xxxxxxAxxx_K_xxxxxxxxxx	11.81	282	2998	20010	327130	1.5
xxxxxxxxxx_K_xxxAxxxxxx	10.40	255	2716	18920	307120	1.5
xxxxxxxxxG_K_xxxxxxxxxx	8.81	254	2461	20326	288200	1.5
xxxxxxxxHx_K_xxxxxxxxxx	9.61	113	2207	7233	267874	1.9
xxxxxxxxxF_K_xxxxxxxxxx	7.82	126	2094	9244	260641	1.7

xxxxxxxxxxY_K_xxxxxxxxxxx	8.41	112	1968	7924	251397	1.8
xxxxxxxxxxE_K_xxxxxxxxxxx	9.07	289	1856	26648	243473	1.4
xxxxxxxxxxx_K_xDxxxxxxxx	7.82	121	1567	9791	216825	1.7
