# Supplementary material for: Quantitative Crotonylome Analysis Reveals the Mechanism of Shenkang Injection on Diabetic Nephropathy
Source: Oxid Med Cell Longev. 2022 Jul 12;2022:7767431. doi: 10.1155/2022/7767431 (PMC11401665; doi:10.1155/2022/7767431)
Supplement: Supplementary 3 — Supplementary Table S2: Molecular docking results of Gpx3 and bioactive constituents of Shenkang injection. [file 7767431.f3.pdf]

Supplementary Table S2. Molecular docking results of Gpx3 and bioactive constituents of Shenkang injection.

Ligand component	Receptor protein	Binding energy (kcal/mol)
Emodin	GPX3	-5.0
Rhein	GPX3	-4.9
Aloe emodin	GPX3	-4.8
Calycosin	GPX3	-4.4
Danshensu	GPX3	-4.0
Gallic acid	GPX3	-3.6
