# Supplementary material for: Quantitative Crotonylome Analysis Reveals the Mechanism of Shenkang Injection on Diabetic Nephropathy
Source: Oxid Med Cell Longev. 2022 Jul 12;2022:7767431. doi: 10.1155/2022/7767431 (PMC11401665; doi:10.1155/2022/7767431)
Supplement: Supplementary 4 — Supplementary Figure S1: Schematic representation of the experimental workflow for tandem mass tag quantification of Kcr in kidney tissues from db/m, db/db, and Shenkang injection-treated db/db mice. [file 7767431.f4.pdf]

Supplementary Figure S1

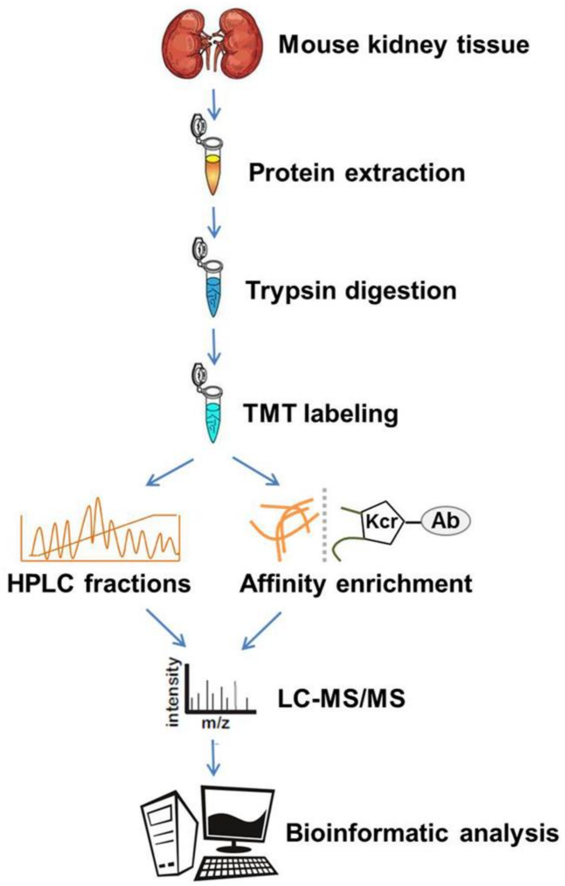

Supplementary Figure S1. Schematic representation of the experimental workflow for tandem mass tag quantification of Kcr in kidney tissues from db/m, db/db, and Shenkang injection treated db/db mice.
