# Supplementary material for: Quantitative Crotonylome Analysis Reveals the Mechanism of Shenkang Injection on Diabetic Nephropathy
Source: Oxid Med Cell Longev. 2022 Jul 12;2022:7767431. doi: 10.1155/2022/7767431 (PMC11401665; doi:10.1155/2022/7767431)
Supplement: Supplementary 5 — Supplementary Figure S2: Verification of the mass spectrometry (MS) data. [file 7767431.f5.pdf]

Supplementary Figure S2

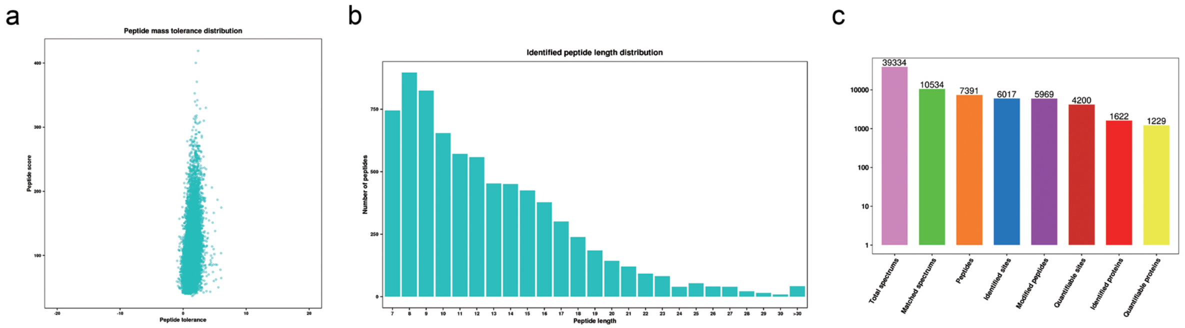

Supplementary Figure S2. Verification of the mass spectrometry (MS) data. (a) Distribution of mass error of all identified crotonylated peptides. (b) Distribution of Kcr peptides based on their length. (c) Overview of Kcr modification identification.
