# Supplementary material for: Quantitative Crotonylome Analysis Reveals the Mechanism of Shenkang Injection on Diabetic Nephropathy
Source: Oxid Med Cell Longev. 2022 Jul 12;2022:7767431. doi: 10.1155/2022/7767431 (PMC11401665; doi:10.1155/2022/7767431)
Supplement: Supplementary 6 — Supplementary Figure S3: The crystal structures of three crotonylated proteins: Ndufs4, cox5a, and Gpx3. [file 7767431.f6.pdf]

Supplementary Figure S3

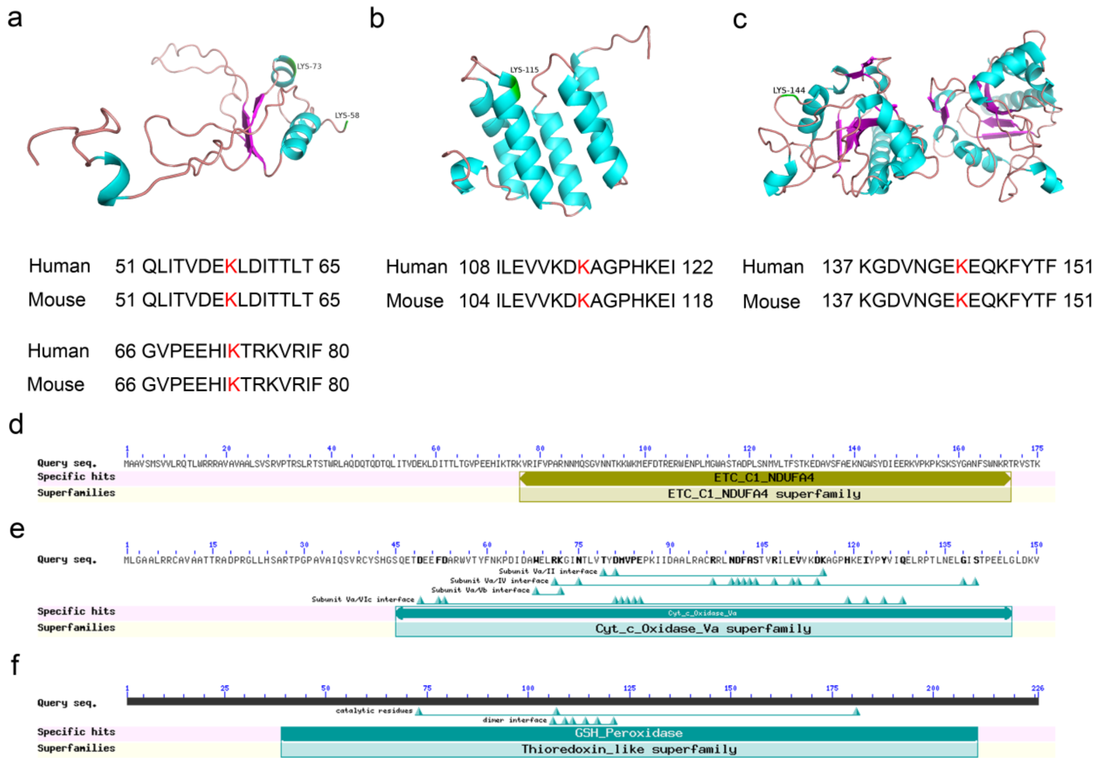

Supplementary Figure S3. The crystal structures of three crotonylated proteins: (a) Ndufs4, (b) cox5a, and (c) Gpx3. Blue and purple represent α helixes and β sheets, respectively. The loop is shown in red. The identified positions of Kcr sites are labeled in green. Structures were generated with PyMOL. Bottom: conservation of the identified Kcr sites from human to mouse. Schematic diagrams of (d) Ndufs4, (e) cox5a, and (f) Gpx3 domains.
