# Supplementary material for: Quantitative Crotonylome Analysis Reveals the Mechanism of Shenkang Injection on Diabetic Nephropathy
Source: Oxid Med Cell Longev. 2022 Jul 12;2022:7767431. doi: 10.1155/2022/7767431 (PMC11401665; doi:10.1155/2022/7767431)
Supplement: Supplementary 7 — Supplementary Figure S4: The 3D interaction diagrams of Gpx3 and bioactive constituents of Shenkang injection. [file 7767431.f7.pdf]

Supplementary Figure S4

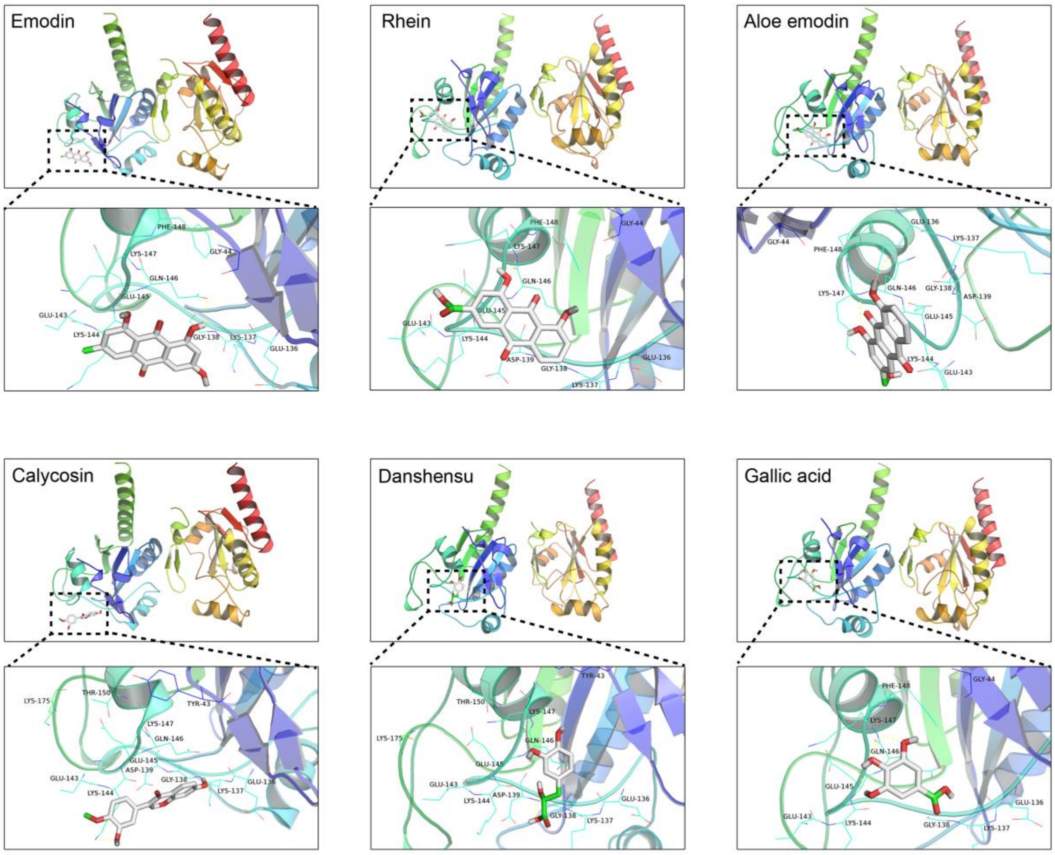

Supplementary Figure S4. The 3D interaction diagrams of Gpx3 and bioactive constituents of Shenkang injection.
